# A Robust Triboelectric Impact Sensor with Carbon Dioxide Precursor-Based Calcium Carbonate Layer for Slap Match Application

**DOI:** 10.3390/mi14091778

**Published:** 2023-09-17

**Authors:** Inkyum Kim, Hyunwoo Cho, Narasimharao Kitchamsetti, Jonghyeon Yun, Jeongmin Lee, Wook Park, Daewon Kim

**Affiliations:** 1 Department of Electronics and Information Convergence Engineering, Kyung Hee University, 1732 Deogyeong-daero, Giheung-gu, Yongin 17104, Republic of Korea; inkyum.kim@khu.ac.kr (I.K.); hyunwoo.cho@khu.ac.kr (H.C.);; 2 Institute for Wearable Convergence Electronics, Kyung Hee University, 1732 Deogyeong-daero, Giheung-gu, Yongin 17104, Republic of Korea; 3 Department of Electronic Engineering, Kyung Hee University, 1732 Deogyeong-daero, Giheung-gu, Yongin 17104, Republic of Korea

**Keywords:** triboelectric nanogenerators, calcium carbonate, triboelectric series, CO_2_ absorption, eco-friendly technology, slap match

## Abstract

As an urgent international challenge, the sudden change in climate due to global warming needs to be addressed in the near future. This can be achieved through a reduction in fossil fuel utilization and through carbon sequestration, which reduces the concentration of CO_2_ in the atmosphere. In this study, a self-sustainable impact sensor is proposed through implementing a triboelectric nanogenerator with a CaCO_3_ contact layer fabricated via a CO_2_ absorption method. The triboelectric polarity of CaCO_3_ with the location between the polyimide and the paper and the effects of varying the crystal structure are investigated first. The impact sensing characteristics are then confirmed at various input frequencies and under applied forces. Further, the high mechanical strength and strong adherence of CaCO_3_ on the surface of the device are demonstrated through enhanced durability compared to the unmodified device. For the intended application, the as-fabricated sensor is used to detect the turning state of the paper Ddakji in a slap match game using a supervised learning algorithm based on a support vector machine presenting a high classification accuracy of 95.8%. The robust CaCO_3_-based triboelectric device can provide an eco-friendly advantage due to its self-powered characteristics for impact sensing and carbon sequestration.

## 1. Introduction

Due to the increased effects of global warming, such as droughts and flooding, the environment is threatened [1]. To solve this problem, numerous efforts have been directed towards reducing the emission of greenhouse gases. The Paris Agreement, which is an international treaty aimed at solving the climate change problem, entered into force in 2016. The objectives of this treaty are to limit the rise in average temperature of the Earth to a maximum of 2 °C and to avoid exceeding a temperature increase of more than 1.5 °C in order to prevent the worst scenario of climate change [2].

A reduction in the usage of fossil fuels would be another approach to decrease the atmospheric concentration of CO_2_. With the increased number of small electronic devices, this could be achieved through implementing self-powered devices which can operate without combusting fossil fuels [3,4,5,6,7]. Energy harvesting technology, which converts ambient energy into electricity, can be applied to this field, and mechanical energy harvesting is particularly well-suited for powering self-powered sensors [8,9,10,11,12,13,14,15]. In this scenario, piezoelectric [16,17,18,19,20,21], electromagnetic [22,23,24], and triboelectric methods [25,26,27] are available for generating electricity from the kinetic energy of physical motion.

The triboelectric nanogenerator (TENG) is a device that has emerged as a promising technology for harvesting kinetic energy from the environment. One of the key advantages of the TENG is its ability to produce high voltage outputs with a relatively simple device structure. The TENG typically consists of three layers: a metal layer, an attached insulating layer, and a separate counter dielectric layer [28,29,30,31]. This structure allows the TENG device to generate a displacement current via contact electrification and electrostatic induction [32]. The TENG has been used for a wide range of applications, including monitoring of wind, rain, and other conditions using the generated electrical output signals [33,34,35,36,37]. An additional noteworthy merit of the triboelectric nanogenerator (TENG) lies in its applicability as a force-responsive sensor. Through calibrating specific benchmarks for electrical outputs, the TENG offers a robust method for quantifying both magnitude and vectorial components of exerted forces [38,39,40,41]. Additionally, the TENG’s ability to generate electrical signals upon minimal mechanical interaction enables the harvesting of low levels of kinetic energy. This feature augments the device’s range of energy-harvesting capabilities, thereby enhancing its overall efficacy.

The above method of energy harvesting can be combined to further contribute to the deceleration of the CO_2_ gas emission rate through fabricating a triboelectric sensor in which the component material is synthesized using a CO_2_ absorption method [42]. In this respect, CaCO_3_ is a suitable target material for the eco-friendly fabrication of a triboelectric impact sensor, owing to its high mechanical robustness [43]. Moreover, due to the hydrophilic characteristics of CaCO_3_ [44], a drop-casting solution can be easily prepared for coating onto a target surface [45,46,47,48]. The ready availability and abundance of CaCO_3_ in nature, coupled with its high biocompatibility, are further advantages of this material [49,50,51].

Notwithstanding these benefits, a comprehensive investigation into the triboelectric properties of CaCO_3_ across varying crystal structures is imperative for broadening the material’s applicability. Elucidating these properties could catalyze the advancement of more efficacious triboelectric sensors, thereby diversifying their potential applications, including but not limited to energy harvesting and environmental surveillance. Triboelectric sensors fabricated with CO_2_-absorbed materials provide a unique solution to mitigate the effects of climate change while generating energy in an eco-friendly manner. The further optimization of the synthesis process and investigation of the characteristics of various crystal structures can facilitate the fabrication of more efficient and effective triboelectric sensors.

In the present paper, a single electrode-based TENG with a triboelectric layer of CaCO_3_ and a controlled surface structure is proposed for use as a self-sustainable triboelectric impact sensor. Two types of CaCO_3_ powder are prepared using CO_2_ absorption and wet chemical synthesis methods. The triboelectric polarity of the CaCO_3_ is examined through using each of four different materials as the counter dielectric layer. For practical utilization, readily available natural CaCO_3_ from eggshell is applied as the contact layer. To use this TENG device as an impact sensor, the frequency, force, and humidity responses are experimentally analyzed through applying mechanical inputs with paper as the counter dielectric layer. The durability of the electrical output is confirmed during the long-term operation of the TENG device.

For the slap match (Ddakji chigi) application [52], each of the five different triboelectric sensors are individually connected to five channels of a multi-channel setup. This slap match game involves attacking one opponent’s Ddakji (defender) with the other opponent’s Ddakji (attacker) in turn. The voltage signals obtained from each channel when an attacker Ddakji device hits a defender Ddakji are processed to judge the turning state of the defender Ddakji. As a user-oriented approach to build a suitable decision boundary, a supervised learning algorithm based on the support vector machine (SVM) is introduced to judge the turning state of the defender Ddakji. This SVM is generally used for binary classification problems, even when high-dimensional data are involved [53], and is reasonably suitable for the present dataset with its small size and low noise [54]. After the training and testing process, the accuracy in judging the rate of turning of the Ddakji is found to be 95.8%. This application of the TENG as a self-sustainable impact sensor with CaCO_3_ as a component material is expected to open up an important eco-friendly strategy.

## 2. Results and Discussion

### 2.1. Synthetic Procedures and Crystallography

**Figure 1:**
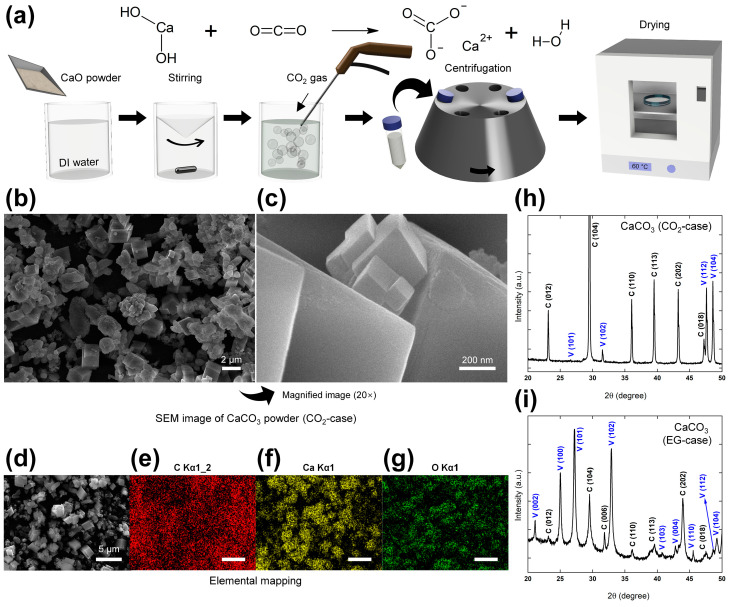
Synthesis and characterization of the CaCO_3_ powder via CO_2_ absorption (the CO_2_-case). (**a**) A schematic diagram showing the synthesis. (**b**,**c**) SEM images at magnifications of (**b**) 10^4^ and (**c**) 2 × 10^5^. (**d**–**g**) EDAX results with the (**d**) surface image, (**e**) carbon map, (**f**) calcium map, and (**g**) oxygen map. (**h**,**i**) XRD spectra for the (**h**) CO_2_-case and (**i**) EG-case.

The fabrication process of the CaCO_3_ powder via a CO_2_ absorption synthesis method is shown in Figure 1a and detailed in the Experimental section. The as-fabricated sample is referred to hereafter as the CO_2_-case. The digital photographic images and ultraviolet-visible (UV-vis) spectra of the initial CaO solution and the obtained CaCO_3_ dispersion are provided in Figure S1 of the Supplementary Materials. There, the CaO solution and CaCO_3_ dispersion exhibit minimum transmittance values of 82.4% and 6.94%, respectively, in the wavelength range of 380–780 nm.

To optimize the CO_2_ gas injection conditions, a series of experiments were conducted employing three distinct gas injection velocities (3.5, 6.0, and 8.5 m s^−1^) and four specific injection durations (7.5, 15, 30, and 60 s). Subsequently, the morphological characteristics of the resultant CaCO_3_ powders were scrutinized to assess the impact of these variables. The results in Figure S2 indicate that the surface structure of the CaCO_3_ increasingly collapses as the velocity and injection time are increased. This is due to the formation of water-soluble Ca(HCO_3_)_2_ according to the following chemical reaction:

CaCO_3_ (s) + H_2_O (l) + CO_2_ (g) → Ca(HCO_3_)_2_ (aq)

With the injection time of 7.5 s, the color of the solution did not change, i.e., CaCO_3_ particles were not formed. Moreover, the velocity of 3.5 m s^−1^ was the minimum value for the CO_2_ gas eruption from the air-gun. Therefore, the conditions of 3.5 m s^−1^ for 7.5 s were selected as the optimum conditions for fabricating the CaCO_3_ powder.

The morphology and microstructure of the CaCO_3_ powder are revealed in the scanning electron microscope (SEM) images in Figure 1b,c, where the presence of a bimodal structure composed of aggregates of smaller-scale (hundreds of nm) cubic crystals measuring under 2 µm can be seen. Furthermore, the SEM images of the commercial CaCO_3_ powder in Figure S3a,b show a similar cubic structure to that of the as-fabricated powder, but the lattice spacing of the commercial powder is larger (over 3 µm). Further, the atomic composition of the as-fabricated CaCO_3_ powder is confirmed by the energy dispersive X-ray spectroscopy (EDAX) and elemental mapping results in Figure 1d–g. Here, the surface of the CaCO_3_ powder specimen is shown in Figure 1d, and the uniform distributions of the elements C, Ca, and O are revealed in the corresponding maps in Figure 1e, Figure 1f, and Figure 1g, respectively.

For comparison, a CaCO_3_ powder with a different crystal structure was prepared via a wet chemical synthesis method [55], as detailed in Figure S4 and the Experimental section. This sample is referred to hereafter as the ethylene glycol (EG)-case. The SEM images of the EG-case are provided in Figure S3c,d, where a bimodal structure consisting of 1–3 µm-diameter spheres and a lattice with tens of nm-scale can be observed. Further, the X-ray diffraction (XRD) spectra of the CO_2_-case, the EG-case, and the commercial product (referred to hereafter as the commercial-case) are presented in Figure 1h, Figure 1i, and Figure S5, respectively. Here, the Miller indices (hkl) are indicated in black text, and the crystallographic planes corresponding to calcite (JCPDS 99-0022) and vaterite (JCPDS 72-0506) are indicated in blue. Thus, the CO_2_-case and commercial-case powders exhibit predominantly the calcite structure, while the EG-case exhibits a mixed vaterite and calcite structure.

### 2.2. Device Preparation

**Figure 2:**
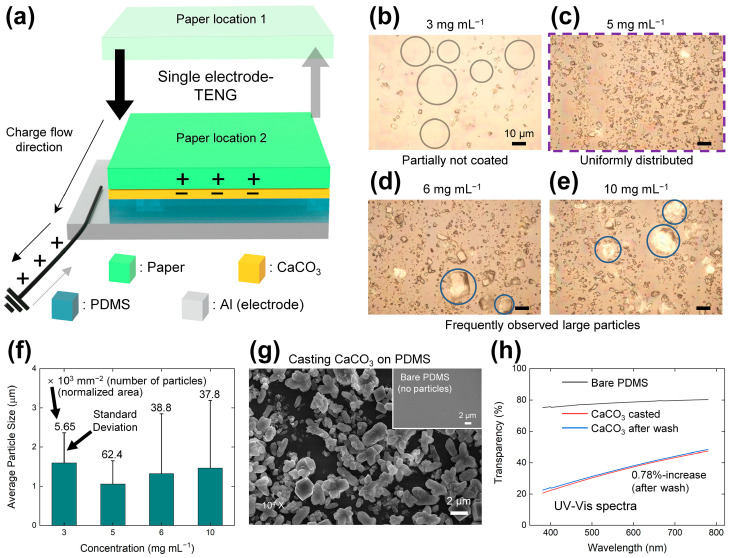
Structure and characterization of the CaCO_3_-based TENG. (**a**) A schematic structure. (**b**–**e**) OM images of the as-cast CaCO_3_ surface using CaCO_3_ concentrations of (**b**) 3 mg mL^−1^, (**c**) 5 mg mL^−1^, (**d**) 6 mg mL^−1^, and (**e**) 10 mg mL^−1^. (**f**) Distribution profile of CaCO_3_ particles with changing concentrations of the dispersion. (**g**) SEM images of the contact surface before (inset) and after casting the CaCO_3_ layer onto the PDMS layer. (**h**) Transparency of the PDMS layer with changing the coating condition.

As shown schematically in Figure 2a, the triboelectric nanogenerator (TENG) device consists of a simple single electrode mode with an electrode layer, a contact layer, and a counter dielectric layer. After a few cycles of contact between the tribo-positive counter dielectric layer and the CaCO_3_ layer, these surfaces become saturated with positive and negative charges, respectively. Then, when the tribo-positive counter dielectric layer approaches the CaCO_3_ layer again, the electrons flow from the ground to the electrode layer via electrostatic induction, while a reverse current is generated when the surfaces are separated. Thus, an alternating current (AC) signal can be generated in one cycle.

The detailed working principle of the TENG is demonstrated in Figure S6. In the initial electrified state, the CaCO_3_ (yellow) and counter dielectric (green) layers are in contact and in electrical equilibrium (Figure S6a). When separation commences, however, the effect of the positive charges at the counter dielectric layer upon the CaCO_3_ layer is reduced, and additional positive charges are induced at the electrode from the ground (Figure S6b). An electrical current can also flow in the same direction as the positive charges. After the separation process, the effect of the counter dielectric layer is removed for the electrical equilibrium at the bottom layer (Figure S6c). Therefore, the number of induced positive charges at the electrode is the same as the number of negative charges at the CaCO_3_ layer. Next, the counter dielectric layer again approaches the CaCO_3_ (Figure S6d), so that the positive charges at the counter dielectric layer can participate in equilibrium with the CaCO_3_ layer, and the previously induced positive charges at the electrode return to the ground. The resulting electrical current at the electrode flows in the reverse direction to that observed during the separating state. As the movement of the counter dielectric layer is repeated, an alternating current (AC) can be generated using the TENG device.

The fabrication process of the TENG device is illustrated in Figure S7a–d and described in the Experimental section. To optimize the drop-casting of the CaCO_3_ layer onto the polydimethylsiloxane (PDMS) layer, various masses of CaCO_3_ powder (3, 5, 6, and 10 mg) were dispersed in ethanol (1 mL). Optical microscopy (OM) images in Figure 2b–e reveal that an optimum concentration of 5 mg mL^−1^ yields maximal uniformity, as substantiated by Figure 2c. In contrast, the circles in Figure 2b indicate vacancies between the CaCO_3_ particles when the concentration is 3 mg mL^−1^, while agglomerated particles of over 10 µm are observed at higher concentrations (circles, Figure 2d,e). Such large particles can lead to point contacts when operating the TENG, thereby decreasing the electrical output. Figure 2f depicts the size distribution profile of the CaCO_3_ particle deposited on the PDMS layer using four different concentrations based on the OM images in Figure 2b–e. The analysis of particle size revealed that the sample with a concentration of 5 mg mL^−1^ exhibited the smallest average value of 1.058 µm, with a standard deviation of 0.597 µm. Additionally, this specific sample exhibited the maximum normalized particle density, attaining a value of 62.4 × 10^3^ mm^−2^. These findings imply a diminished propensity for particle aggregation, coupled with an enhanced spatial distribution of the CaCO_3_ particles in the 5 mg mL^−1^-sample. Additionally, the respective histograms depicting the size distribution of the CaCO_3_ particles can be observed in Figure S8a–d. The optimal concentration is characterized by reduced aggregation and lower viscosity, facilitating the uniform distribution of powder on the PDMS layers [56,57]. An SEM image of the optimally drop-casted CaCO_3_ on the PDMS layer is presented in Figure 2g, where the 2 µm particles are seen to entirely coat the PDMS layer. For comparison, an SEM image of the bare PDMS surface is presented in the inset of Figure 2g, where a smooth surface is observed with no particles. The transparency of the composite layers was measured using a UV-vis spectrophotometer, and the results are presented in Figure 2h. The transparency decreased after coating CaCO_3_ particles onto the PDMS layer. These CaCO_3_ particles remained attached even after the washing process, resulting in only a 0.78% increase in transparency. The attachment of the CaCO_3_ particles can be further confirmed through the UV-vis spectra. The stability of the CaCO_3_ dispersion was evaluated through UV-vis spectroscopy, as illustrated in Figure S9, with a focus on varying stabilization times. After 50 min, the transparency value at a wavelength of 550 nm reached a stable point at 22%. The rate of change in transparency was determined to be 0.32% per min at the saturation point.

### 2.3. Electrical Outputs and Triboelectric Polarity

**Figure 3:**
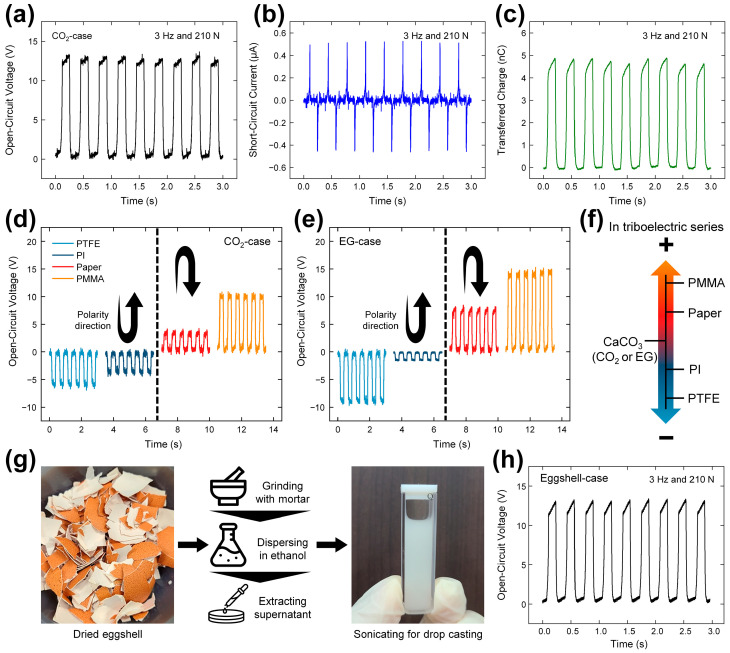
(**a**–**c**) Electrical outputs of the CaCO_3_-based TENG with the CO_2_-case contacting the paper layer: (**a**) *V*_OC_, (**b**) *I*_SC_, and (**c**) *Q*_SC_. (**d**,**e**) Experimental *V*_OC_ results for determining the triboelectric polarity of the CaCO_3_ in the (**d**) CO_2_-case and (**e**) EG-case with various counter dielectric layers. (**f**) Location of CaCO_3_ in the simplified triboelectric series. (**g**) Fabrication of the eggshell-based CaCO_3_ powder. (**h**) *V*_OC_ of the TENG with the contact of the eggshell-based CaCO_3_ layer and paper layer.

The electrical outputs of the TENG fabricated using the CO_2_-case powder are displayed in Figure 3a–c under the mechanical input conditions of 3 Hz and 210 N. Paper was used as the contact layer because the Ddakji used for the intended application is made of filter paper. The open-circuit voltage (*V*_OC_), short-circuit current (*I*_SC_), and short-circuit charge (*Q*_SC_) each exhibit pulse-like waveforms, with peaks of 13.4 V, 0.48 µA (AC), and 4.8 nC, respectively. Given a contact area of 4 cm^2^, the current density value can be calculated as 1.2 mA m^−2^.

To identify the position of CaCO_3_ in the triboelectric series, four distinct materials, namely polytetrafluoroethylene (PTFE, 200 µm), polyimide (PI, 125 µm), filter paper (140 µm), and poly(methyl methacrylate) (PMMA, 2 mm), were separately used as the counter dielectric layer in contact with the CaCO_3_ layer. For both the CO_2_-case and the EG-case, the results in Figure 3d and Figure 3e respectively indicate one electrical output polarity when PTFE or PI is used, and the opposite polarity when paper or PMMA is used. Given that these four materials occupy the triboelectric series in the order of PTFE, PI, paper, and PMMA according to increasing (negative to positive) polarity (Figure 3f) [26,58], CaCO_3_ can be located between the paper and the PI. These results, along with the *I*_SC_ results that shown in Figure S10, further confirm the successful casting of the CaCO_3_ particles onto the PDMS.

To further demonstrate the applicability of the CaCO_3_-based TENG, a device was prepared using a natural material (eggshell) as the source of CaCO_3_, as detailed in Figure 3g and Figure S11a and the Experimental section. The results in Figure 3h and Figure S11b,c indicate comparable electrical outputs to those of the CO_2_-case, with a *V*_OC_ of 13.2 V, an *I*_SC_ of 0.45 µA, and a *Q*_tr_ of 4.89 nC.

### 2.4. The Variation in Electrical Output under Various Conditions

**Figure 4:**
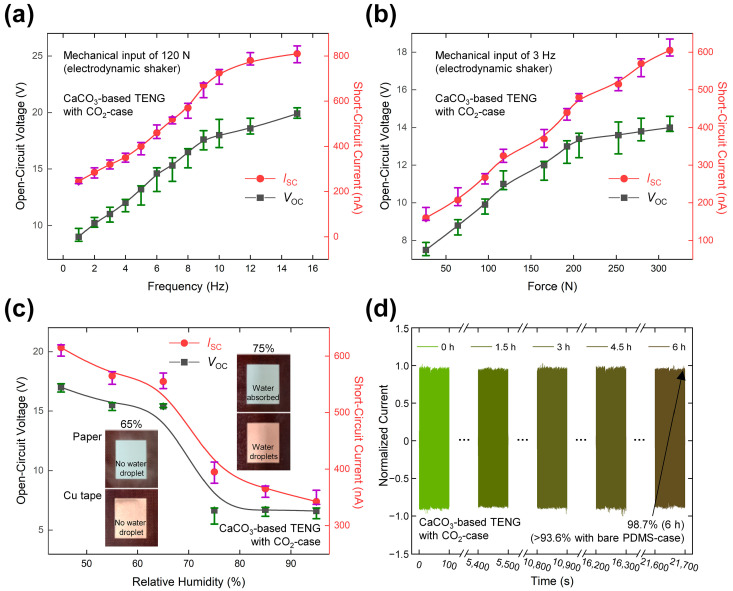
(**a**) A frequency response and (**b**) a force response of the CO_2_-case TENG in terms of the *V*_OC_ (green) and *I*_SC_ (red). (**c**) Corresponding humidity response, with inset digital photographs of the paper and Cu surfaces at 65% and 75% relative humidity. (**d**) Normalized current results from the durability test of the CO_2_-case TENG during 21,700 s of operation.

To examine the use of the CaCO_3_-based TENG as a self-sustainable sensor, the electrical outputs were studied under various input or environmental conditions. When the frequency of the mechanical input was varied between 1 and 15 Hz, the results in Figure 4a indicate that both the *V*_OC_ (left-hand axis, green points) and *I*_SC_ (right-hand axis, red points) of the CO_2_-case TENG increased with increasing input frequency. This can be attributed to the faster flow of charge between the electrode and ground due to the higher operating speed. Moreover, the relative increment in the *I*_SC_ value between 1 and 15 Hz (331%) is larger than that of the *V*_OC_ (221%) due to the same reason. Achieving a complete open-circuit state is challenging with the utilized measuring equipment, and there exists the possibility of charge transfer between the electrode and ground under practical conditions [59]. In addition, the frequency response is affected by the increased impact according to the higher variation in input velocity (∆*ν*) at higher frequency values. Further, as shown in Figure S12a, the EG-case TENG exhibits a similar frequency response to that of the CO_2_-case TENG under the same conditions, but with slightly increased peak values due to the enlarged contact area provided by the nanoscale surface structures (Figure S3d).

The force response of the CO_2_-case TENG was analyzed as detailed in the Experimental section, and the results are presented in Figure 4b. Upon increasing the applied force, the interfacial area between the CaCO_3_ and paper layers expands, resulting in a concurrent augmentation in both *V*_OC_ and *I*_SC_ parameters [60,61]. Furthermore, attributed to a significant enhancement in contact velocity due to simultaneous increments in acceleration and force, the *I*_SC_ demonstrates a relative increase of 378% within the force range of 30–310 N. In contrast, the *V*_OC_ shows a more modest increase of 187%. The observed plateauing of *V*_OC_ at applied forces exceeding 210 N can be attributed to the repulsive forces exerted by the underlying PDMS layer, which act to limit any further extension in the contact area [62]. For comparison, the force response of the EG-case TENG is presented in Figure S12b, where a similar trend is observed. These results exhibit a maximum error rate of 13.9%.

The humidity response was measured as described in the Experimental section, and the results are presented in Figure 4c. Here, the *V*_OC_ and *I*_SC_ of the CO_2_-case TENG are observed to decrease sharply to 43 and 78%, respectively, at relative humidity (RH) values of 65–75%. This phenomenon can be ascribed to the cohesive interaction of water molecules on the contact interface within this RH range, inhibiting complete interfacing and subsequent triboelectrification between the CaCO_3_ and paper layers at elevated RH values. This is further demonstrated by the digital photographs of the paper and Cu tape in the inset of Figure 4c. Water droplets are not observed on the surface of either film at an RH of 65% but are clearly visible on the Cu tape and the ground wood at 75% RH. However, due to its water absorbing characteristics, the paper remains in the same state regardless of the RH [63,64]. The same tendency is observed for the EG-case TENG in Figure S12c. These results exhibit an error rate of under 12.7%, thus enabling the impacting condition of the CaCO_3_-based TENG to be sensed with proper calibration according to the environmental conditions.

The output power values of the CO_2_-case and EG-case TENGs are presented in Figure S13a and S13b, respectively. These were calculated using Equation (1):

*P* = *I*^2^*R* (1)

where *P*, *I*, and *R* represent the output power, output current, and load resistance, respectively. The output current was measured through connecting the CaCO_3_-TENG device to a series load resistor. Under the conditions of 3 Hz and 210 N, the peak value of output current decreased with increasing load resistance in accordance with Ohm’s law. Thus, the calculated maximum output power values of the CO_2_-case and EG-case TENGs were 15.19 µW (37.97 mW m^−2^) at 300 MΩ and 20.58 µW (51.46 mW m^−2^) at 350 MΩ, respectively. The higher output power of the EG-case can be attributed to the large contact area provided by the nanoscale surface structures on the CaCO_3_ powder. The energy harvesting efficiency of the CO_2_-case TENG is determined by the ratio of the output power to the product of the input force and the displacement of the input component. An energy harvesting efficiency from the device with 0.00145% is achievable with output power, input force, and input displacement values of 15.19 µW, 210 N, and 5 mm, respectively.

To demonstrate the durability of the CaCO_3_-TENG device, a long-term endurance test was conducted through measuring *I*_SC_ during 21,700 s, and the result was compared with that of a single electrode-based TENG that was prepared without a CaCO_3_ layer (designated the bare PDMS-case). The result for the bare PDMS-case device is provided in Figure S14, and that for the CO_2_-case device is presented in Figure 4d. For ease of comparison, the *I*_SC_ was normalized to a larger peak value during the initial cycle. The results indicate that the normalized *I*_SC_ of the CO_2_-case device is only decreased to 0.987 after 21,700 s of operation, compared to 0.936 for the bare PDMS-case device, this demonstrating the superior durability of the CO_2_-case device. Figure S15a shows the presence of CaCO_3_ particles coating the PDMS surface following the measurement. The OM image in Figure S15a reveals a decrease in the average particle size to 0.934 µm, with a corresponding standard deviation of 0.246 µm, compared to the pre-measurement samples shown in Figure 2c,f. Additionally, the OM image in Figure S15a exhibits an increased normalized number of particles, quantified as 124.3 × 10^3^ mm^−2^, indicating the occurrence of particle fragmentation during the measurement. The size distribution of the particles in the OM image can be observed in the histogram presented in Figure S15b. This is attributed to both the high mechanical strength of the CaCO_3_ and the well-adhered state of the CaCO_3_ powders on the PDMS layer. These results further demonstrate that the CaCO_3_-based TENG can serve a practical role as an impact sensor.

### 2.5. Application of the Device for the Slap Match Game

For use in playing a slap match game, the as-fabricated CaCO_3_-based TENG was applied as an impact sensor. Five impact sensors, including four CO_2_-case sensors and one EG-case sensor, were connected to one channel each of a multi-channel setup to determine the attacking location and intensity of each sensor. Owing to its augmented output, the previously mentioned EG-case impact sensor was strategically positioned at the central location and interfaced with Channel 1 (Ch1), thereby enhancing its sensitivity for detecting central attacks. The four CO_2_-case impact sensors were located at the left, top, right, and bottom positions, and connected to Ch2–5.

**Figure 5:**
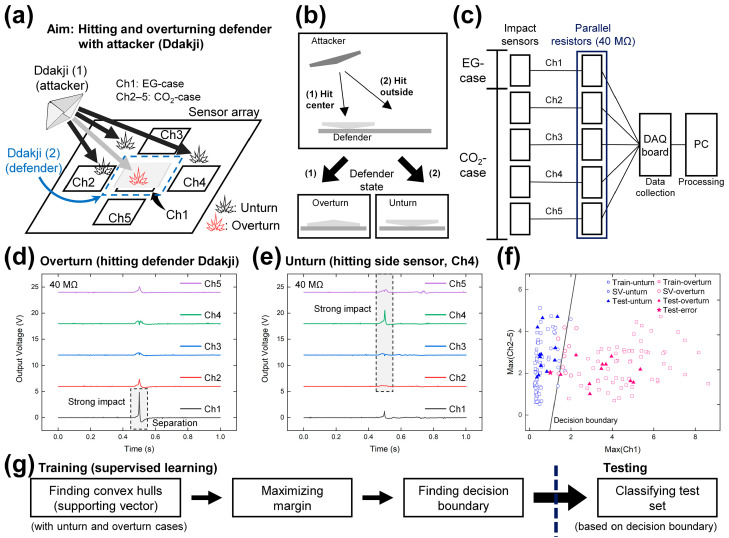
Demonstration of the application of the as-fabricated TENG in a slap match game. (**a**) A schematic illustration of the slap match game and triboelectric impact sensor layout. (**b**) Turning mechanism for Ddakji with two different hitting points. (**c**) A block diagram showing the connections between the impact sensors and the processor. (**d**,**e**) Output voltage results from the impact sensor array in (**d**) the overturned case and (**e**) unturned case. (**f**) SVM classification results for identifying the overturned and unturned cases. (**g**) A training/testing process for classifying the turning state of Ddakji using SVM.

The slap match game involves turning the defender Ddakji (2) through hitting it with the attacker Ddakji (1), as shown in Figure 5a. If the opponent’s defender Ddakji is overturned after being hit, the proponent wins the game. However, if the opponent’s defender Ddakji remains unturned, the opponent has a chance to use his attacker Ddakji to hit the proponent’s defender Ddakji. This process is repeated until a winner emerges. Each Ddakji was fabricated through origami using two pieces of filter paper, as shown in Figure S16. The thickness of the attacker Ddakji was increased through raising the height of the initial paper and flipping it to match the height of the thin Ddakji, as depicted in Figure S16. To enhance the ease of turning, the thin defender Ddakji was positioned in the upside-down state, revealing its reverse side to the observer, as illustrated in Figure 5b. When the thick attacker Ddakji hits the center of the defender Ddakji (Ch1), the defender Ddakji will be overturned (state 1 in Figure 5b). However, if the attacker Ddakji lands outside of the defender Ddakji, the latter will remain unturned (state 2 in Figure 5b). The overturned and unturned states are also illustrated in Figure 5a.

The configuration of the detecting system for the slap match game is illustrated in Figure 5c. Each of the five impact sensors was connected to one channel of the DAQ board via the parallel resistor. The 40 MΩ-resistor was experimentally selected to attain a stable baseline and to generate a suitably high output voltage. The output voltage signals from the five impact sensors were transferred to the PC for data processing.

The representative output signals obtained at each channel when the attacker Ddakji makes a direct hit and overturns the defender Ddakji are shown in Figure 5d and Video S1, while those obtained in the unturned case are shown in Figure 5e and Video S2. In the overturned case (Figure 5d), an intense positive peak voltage followed by a weak negative peak can be detected at Ch1. The positive peak represents the stronger contact between the defender Ddakji and the central EG-case impact sensor, while the negative peak indicates the separation of the defender Ddakji from the central impact sensor. In the unturned case, however, the highest peak voltage is detected at the channel connected to the CO_2_-case impact sensor that is hit by the attacker (Ch4 in Figure 5a). This positive peak is lower than that of the peak observed in the overturned state because the CO_2_-case sensor generates a lower electrical output than the EG-case sensor. The subsequent negative peak voltage is also lower for the CO_2_-case impact sensor (Figure 5e) compared to the EG-case sensor (Figure 5d) due to the brief amount of time needed for contact electrification between the attacker Ddakji and the CO_2_-case impact sensor.

Data comprising 80 unturned cases and 80 overturned cases were amassed and subsequently partitioned into 136 and 24 data points, designated for the training and test sets, respectively. These were then labelled to be applied for the support vector machine (SVM) as a supervised learning algorithm for binary classification, as detailed in the Experimental section. Figure 5f shows the plotted datapoints for the 80 unturned cases (blue) and 80 overturned cases (pink) that were divided into the training data (squares), support vectors (circles), and test data (triangles and star) in two dimensions (2D). The star represents the error after classifying the test set. The decision boundary is indicated by the black line. The *x*-axis and *y*-axis of this graph represent the maximum value from Ch1 and Ch2–5, respectively, which are the parameters to be classified using the SVM. The test data that are located above (to the left of) the decision boundary can be classified as the unturned case, and those located under (to the right) of the decision boundary can be classified as the overturned case.

The flow of classification is displayed in Figure 5g. Convex hulls corresponding to both unturned and overturned cases were identified to optimize the decision boundary, aiming to maximize the margin between the two respective hulls. The optimized decision boundary was subsequently employed for data classification within the test set and for the evaluation of classification accuracy. To examine the effect of the number of dimensions on the SVM classification results, both five dimensions (5D) and 2D were used. Further, to demonstrate the capability of the SVM for high dimension classification, the maximum values from the five channels were directly used in the 5D case. In the 2D case, the two maximum values from Ch1 and Ch2–5 were used to visualize the classification result on a 2D graph. In both the 5D and 2D cases, the test set provided a classification accuracy of 95.8% (23/24). With three additional values from Ch2–5 (excluding the maximum value), the test accuracy of the 5D case was 97.1% (132/136), which was slightly higher than that of the 2D case, i.e., 96.3% (131/136). With these high accuracy values for classification, the applicability of the TENG based on the mechanically robust CaCO_3_ and fabricated via either the CO_2_ absorption or wet chemical synthesis method was successfully demonstrated through this slap match game.

## 3. Conclusions

In this report, CaCO_3_-based triboelectric nanogenerators (TENGs) with two different crystal structures were prepared via CO_2_ absorption and wet chemical synthesis methods (designated the CO_2_-case and the EG-case, respectively) for application as an impact sensor. The X-ray diffraction (XRD) results indicated that the EG-case powder had a combination of calcite and vaterite structures, while the CO_2_-case was predominantly calcite. After optimization of the CO_2_ injecting conditions, the dispersions of each CaCO_3_ powder were drop-cast onto polydimethylsiloxane (PDMS) substrates. The electrical output performance of the as-fabricated CaCO_3_-based TENG devices was empirically validated. Furthermore, the triboelectric polarity of CaCO_3_ was ascertained to reside intermediate to that of polyimide (PI) and paper within the triboelectric series. The practical applicability of the TENG fabricated using eggshell-based CaCO_3_ powder was successfully demonstrated. The characteristics of the TENG for use as an impact sensor were examined through determining the trends in the open-circuit voltage and short-circuit current in the frequency and force responses. Moreover, the enhanced durability due to the mechanically robust and well-adhered CaCO_3_ particles on the PDMS layer was demonstrated by the normalized short-circuit current of 0.987 after operation for 21,700 s. Finally, the application of the as-fabricated TENG device was demonstrated in a slap match game in which the turning state of the defender Ddakji was detected using a TENG impact sensor array connected to a multi-channel setup. The support vector machine was used as a supervised learning algorithm to analyze the data from 80 unturned and 80 overturned cases with 95.8% of classification accuracy. The application of the biocompatible CaCO_3_-based TENG as a wearable device will be discussed in future work.

## 4. Experimental

### 4.1. Chemicals and Materials

Calcium oxide (CaO; reagent grade, powder), calcium carbonate (CaCO_3_; ACS reagent, powder; ACS), and sodium carbonate (Na_2_CO_3_; ACS reagent, powder) were purchased from Sigma-Aldrich (Burlington, MA, USA). Carbon dioxide (CO_2_) gas with a purity of >99.0% was used to synthesize CaCO_3_ via the CO_2_ absorption method. Calcium chloride (CaCl_2_; extra pure, powder) and ethylene glycol (EG; extra pure) were purchased from Daejung Chemicals & Metals Co., Ltd. (Siheung, Republic of Korea). Qualitative filter paper No. 1 was purchased from Whatman (Kent, UK).

### 4.2. Preparation of the CaCO_3_ Powders

CO_2_-case: A dispersion of 1 M calcium oxide (CaO) powder (2 mL) was added to 160 mL of deionized (DI) water and mixed using a magnetic stirrer at 500 rpm for 10 min until a transparent solution was obtained. Then, CO_2_ gas was injected into the solution through an air-gun at a pressure of 0.1 MPa for about 10 s to achieve a saturated opaque solution. After centrifugation at 12,000 rpm for 2 min, the collected particles were stored in ethanol and dried overnight at 60 °C to obtain the CaCO_3_ powder.

EG-case: The calcium chloride (CaCl_2_) and sodium carbonate (Na_2_CO_3_) precursors were dissolved in a mixture of ethylene glycol (EG) and DI water in a volume ratio of 5:1 and stirred for 60 min. This was followed by centrifugation at 5000 rpm for 2 min, with the collected powders being stored in ethanol. Subsequently, the CaCO_3_ powder was dried overnight in a convection oven at 60 °C.

### 4.3. Preparation of the PDMS Layer

Polydimethylsiloxane (PDMS) with Sylgard 184 manufactured by Dow Silicones (Corporation, MI, USA) were coated onto the Al electrode layer (Figure S7a). The base and curing agent were mixed in a weight ratio of 10:1, then placed in a vacuum chamber to remove any gases present. The as-prepared PDMS was then poured onto an Al layer that was affixed to a Si wafer with polyimide (PI) tape. The poured PDMS layer was spin-coated at 750 rpm for 10 s before curing in a convection oven at 60 °C for 6 h. The thickness of the cured PDMS layer was determined at four distinct points using vernier calipers, yielding an average value of 156.75 µm.

### 4.4. Drop-Casting of the CaCO_3_ Layer

The CaCO_3_ powder (3, 5, 6, and 10 mg) was dispersed in ethanol (1 mL) and drop-cast onto the as-prepared PDMS layer. After determining the optimum concentration via optical microscopy (OM), this dispersion (5 mg mL^−1^) was drop-cast at 60 °C for 5 min using a hotplate (Figure S7b). To enhance the uniformity of the cast CaCO_3_ layer, the layer was hand-wiped six to eight times using a laboratory wiper (Figure S7c) and washed with DI water in series (Figure S7d). The fabrication of the TENG device was then completed through attaching electrical wiring to the electrode under the taped part.

To demonstrate further the applicability of the CaCO_3_-based TENG, another device was prepared using a natural material (eggshell) as the source of CaCO_3_, as shown in Figure 3g and Figure S11a. The dried eggshell was heated at 65 °C overnight and then ground in a mortar for 30 min. After grinding, the powder (20 mL) was dispersed in 80 mL of ethanol and sonicated for 6 min. The supernatant was collected and used for drop-casting using a similar process to that employed in the fabrication of the other TENGs (Figure S7).

### 4.5. Characterization of the CaCO_3_

Scanning electron microscope (SEM) images and energy-dispersive X-ray spectroscopy analysis (EDAX) data were collected using a high-resolution field emission SEM (HR FE-SEM), MERLIN manufactured by Carl Zeiss (Oberkochen, Germany) under the conditions of 10 kV and 3.1–5.2 mm. An X-ray diffractometer (XRD), SmartLab manufactured by Rigaku Corporation (Tokyo, Japan) with a power of 3 kW was used to confirm the crystal structures of the as-prepared and purchased CaCO_3_ powders. The color of the solution/dispersion was examined using an ultraviolet-visible (UV-vis) spectrophotometer, LAMBDA™ 365 manufactured by PerkinElmer Inc. (Waltham, MA, USA). To optimize the concentration of the CaCO_3_ dispersion, an optical microscope, Eclipse LV100 manufactured by Nikon Corporation (Tokyo, Japan) with a 100× objective lens and a 3-megapixel digital camera was used.

### 4.6. Measurement of the Electrical Output from the TENG

Force was applied via an electrodynamic shaker, LW139.138-40 manufactured by Labworks Inc. (Costa Mesa, CA, USA) controlled by the signal from a function generator, 33120A manufactured by Agilent Technologies, Inc. (Santa Clara, CA, USA) under various input forces and frequencies. A system electrometer, Model 6514 manufactured by Keithley Instruments (Solon, OH, USA) was used to collect the electrical output data using a multi-channel DAQ system, PCI-6220 manufactured by National Instruments (Austin, TX, USA). A force sensor, 1053v4 manufactured by Dytran Instruments, Inc. (Chatsworth, CA, USA) and an amplifier, E4110C manufactured by Dytran Instruments, Inc. (Chatsworth, CA, USA) were used to measure and visualize the applied force using an oscilloscope, TDS2012B manufactured by Tek Instruments (Beaverton, OR, USA). For the slap match application, a 3D printer, 3DP-310F manufactured by Cubicon (Seongnam, Republic of Korea) was used to form a 10 cm × 10 cm impact sensor array with 1 cm gaps between neighboring sensors on printed acrylonitrile butadiene styrene (ABS, ABS-A100). The force response of the TENG was evaluated through measuring the peak *V*_OC_ and *I*_SC_ values at a fixed frequency of 3 Hz, assuming that each individual contact/separation cycle lasts 0.33 s. The humidity response study was performed using a humidifier to alter the atmospheric conditions of the surroundings and measuring the electrical output under the input conditions of 3 Hz and 210 N.

### 4.7. Coding for the Slap Match Application

The real-time visualization of the signals from the five channels of the DAQ system was made available using LabVIEW coding. The analysis using the support vector machine (SVM) was conducted through reading the collected raw data with Python code and using the svm.SVC term in the scikit-learn library. The 5-dimensional (5D) data were directly analyzed using the SVM, but 2-dimensional (2D) data were passed through a dimension-decreasing process via extracting the maximum value from channels 2–5 (Ch2–5). The regulation parameter I and kernel coefficient (gamma) for the SVM classification were varied to achieve the highest classification accuracy. The optimized regulation parameters for the 5D and 2D cases were 1.2 and 1.0, respectively. The kernel coefficient was fixed in the ‘auto’ state, which uses the value of 1/(number of features).

## Data Availability

Not applicable.

## Supplementary Materials

The following supporting information can be downloaded at: https://www.mdpi.com/article/10.3390/mi14091778/s1, Figure S1: UV-vis spectra with CaO solution and CaCO_3_ dispersion; Figure S2: Surface structure of CaCO_3_ particles with varying fabricating conditions; Figure S3: Surface structures of two different CaCO_3_ powders with commercial-case and EG-case; Figure S4: Synthetic process for CaCO_3_ powder with EG-case; Figure S5: XRD spectrum to check the crystal structure of commercial CaCO_3_ powder; Figure S6: Working principle of the fabricated TENG; Figure S7: Fabrication process for CaCO_3_-based TENG device; Figure S8: Size distribution of the CaCO_3_ particles with four different concentrations of dispersion; Figure S9: Dispersion stability result of the CaCO_3_ powder in ethanol; Figure S10: ISC curves to check the triboelectric polarity of CaCO_3_; Figure S11: Fabrication process of eggshell-based CaCO_3_ and electrical outputs using this powder; Figure S12: Electrical output-response results from CaCO_3_-based TENG with EG-case; Figure S13: Output current and power curves of two CaCO_3_-based TENGs; Figure S14: Durability result of TENG with a bare PDMS layer; Figure S15: Surface and size distribution of the CaCO_3_ particles after measurement; Figure S16: Fabrication process of Ddakji for slap match application. Video S1: Video of overturn case in slap match game; Video S2. Video of unturn case in slap match game.
