# Supplementary material for: A Robust Triboelectric Impact Sensor with Carbon Dioxide Precursor-Based Calcium Carbonate Layer for Slap Match Application
Source: Micromachines (Basel). 2023 Sep 17;14(9):1778. doi: 10.3390/mi14091778 (PMC10537528; doi:10.3390/mi14091778)
Supplement: Supplementary file 1 [file micromachines-14-01778-s001.zip › micromachines-2601399-supplementary/[20230916] (SM) CaCO3-TENG.pdf]

Supplementary Materials

A Robust Triboelectric Impact Sensor with Carbon Dioxide Precursor-Based Calcium Carbonate Layer for Slap Match Application

Inkyum Kim [1,2,†], Hyunwoo Cho [1,2,†], Narasimharao Kitchamsetti [2,3], Jonghyeon Yun [1,2], Jeongmin Lee [3], Wook Park [1,2,*] and Daewon Kim [2,3,*]

[1] Department of Electronics and Information Convergence Engineering, Kyung Hee University, 1732 Deogyeong-daero, Giheung-gu, Yongin 17104, Republic of Korea; inkyum.kim@khu.ac.kr (I.K.); hyunwoo.cho@khu.ac.kr (H.C.); jonghyeon.yun@khu.ac.kr (J.Y.)

[2] Institute for Wearable Convergence Electronics, Kyung Hee University, 1732 Deogyeong-daero, Giheung-gu, Yongin 17104, Republic of Korea; kitchamsetti.rao@khu.ac.kr (N.K.)

[3] Department of Electronic Engineering, Kyung Hee University, 1732 Deogyeong-daero, Giheung-gu, Yongin 17104, Republic of Korea; jml289@khu.ac.kr (J.L.)

* Correspondence: parkwook@khu.ac.kr (W.P.); daewon@khu.ac.kr (D.K.)

† These authors contributed equally to this work.

Table of contents

1. **UV-vis spectra with CaO solution and $CaCO_3$ dispersion**
2. **Surface structure of $CaCO_3$ particles with varying fabricating conditions**
3. **Surface structures of two different $CaCO_3$ powders with commercial-case and EG-case**
4. **Synthetic process for $CaCO_3$ powder with EG-case**
5. **XRD spectrum to check the crystal structure of commercial $CaCO_3$ powder**
6. **Working principle of the fabricated TENG**
7. **Fabrication process for $CaCO_3$-based TENG device**
8. **Size distribution of the $CaCO_3$ particles with four different concentrations of dispersion**
9. **Dispersion stability result of the $CaCO_3$ powder in ethanol**
10. **I_{SC} curves to check the triboelectric polarity of $CaCO_3$**
11. **Fabrication process of eggshell-based $CaCO_3$ and electrical outputs using this powder**
12. **Electrical output-response results from $CaCO_3$-based TENG with EG-case**
13. **Output current and power curves of two $CaCO_3$-based TENGs**
14. **Durability result of TENG with a bare PDMS layer**
15. **Surface and size distribution of the $CaCO_3$ particles after measurement**
16. **Fabrication process of Ddakji for slap match application**
17. **Video of overturn case in slap match game**
18. **Video of unturn case in slap match game**

1. UV-vis spectra with CaO solution and $CaCO_3$ dispersion

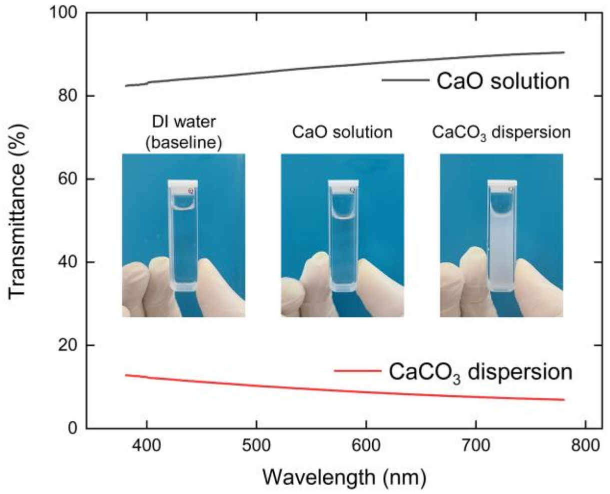

Figure S1. Spectra measured by the UV-vis spectrophotometer with the CaO solution and $CaCO_3$ dispersion. Digital camera images of the DI water (for baseline), CaO solution, and $CaCO_3$ dispersion in the insets.

2. Surface structure of $CaCO_3$ particles with varying fabricating conditions

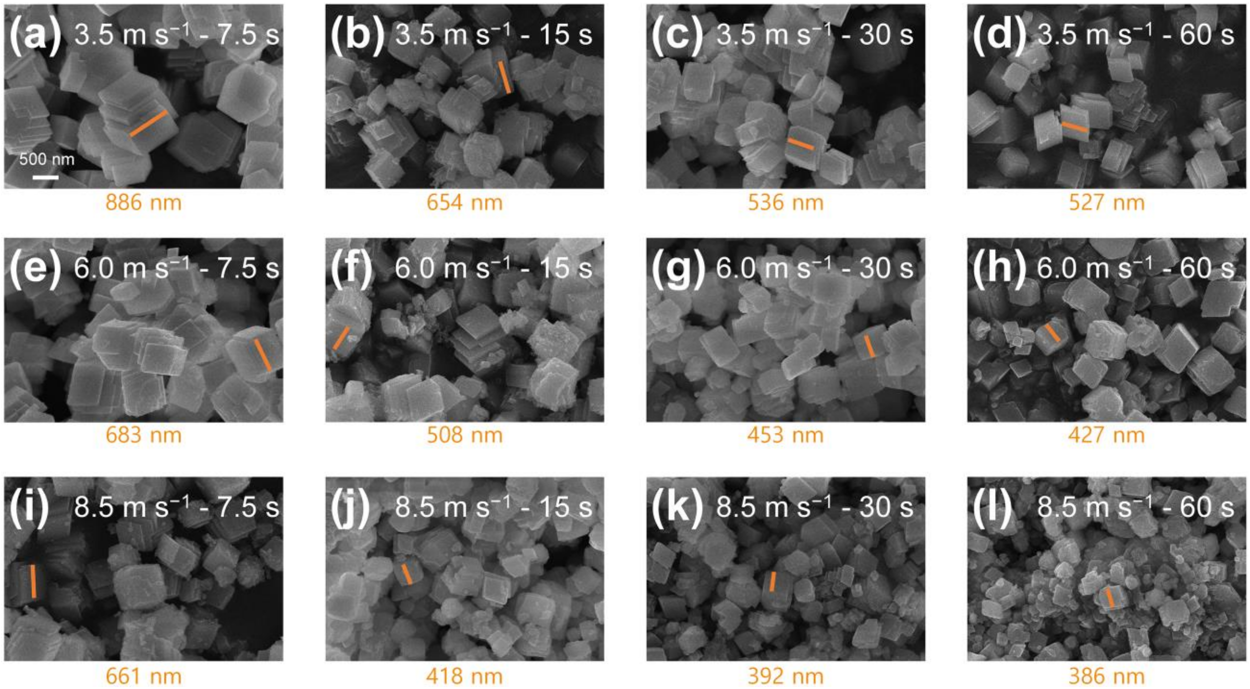

Figure S2. SEM images with varying the fabricating conditions for $CaCO_3$ powder. (a–d) CO_2 gas injection velocity of 3.5 m s^{-1} while changing the injection time with 7.5, 15, 30, and 60 s, respectively. (e–h) CO_2 gas injection velocity of 6 m s^{-1} with changing the injection time with 7.5, 15, 30, and 60 s, respectively. (i–l) CO_2 gas injection velocity of 8.5 m s^{-1} with changing the injection time with 7.5, 15, 30, and 60 s, respectively.

3. Surface structures of two different $CaCO_3$ powders with commercial-case and EG-case

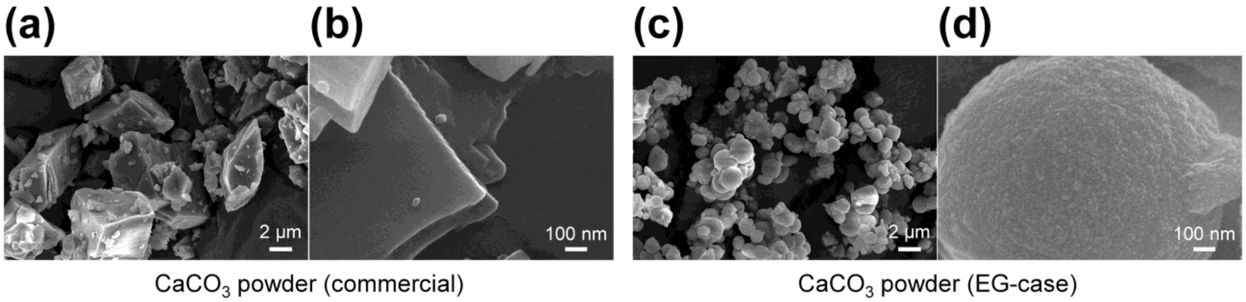

Figure S3. SEM images of two $CaCO_3$ powders: (a) commercial-case with the magnitude of 10^4, (b) commercial-case with the magnitude of 2×10^5, (c) EG-case with the magnitude of 10^4, and (d) EG-case with the magnitude of 2×10^5.

4. Synthetic process for $CaCO_3$ powder with EG-case

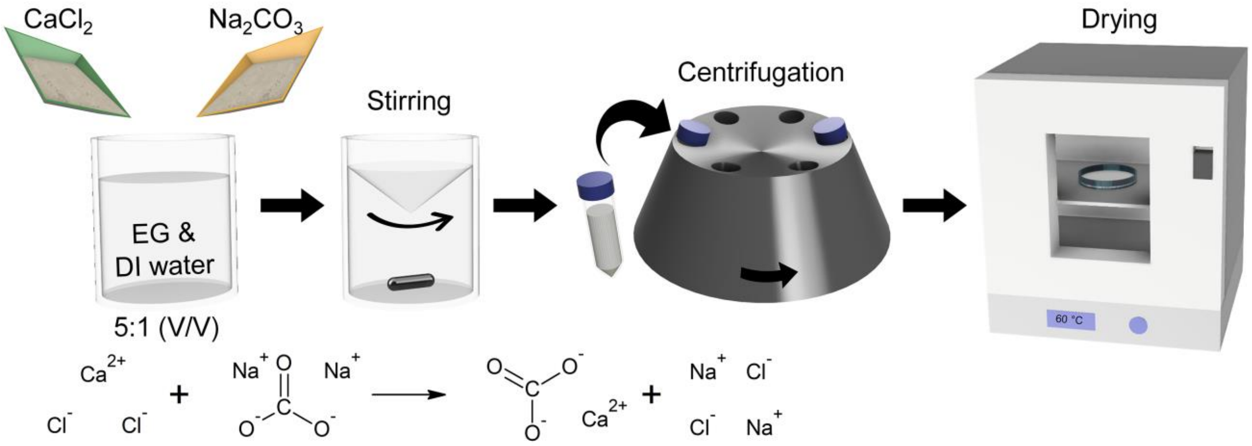

Figure S4. Wet chemical synthesis method for the $CaCO_3$ powder (with the EG-case).

5. XRD spectrum to check the crystal structure of commercial $CaCO_3$ powder

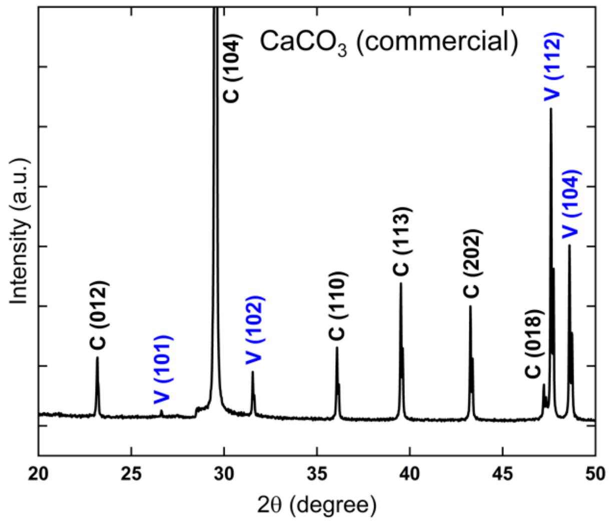

Figure S5. XRD spectrum of the commercial $CaCO_3$ powder.

6. Working principle of the fabricated TENG

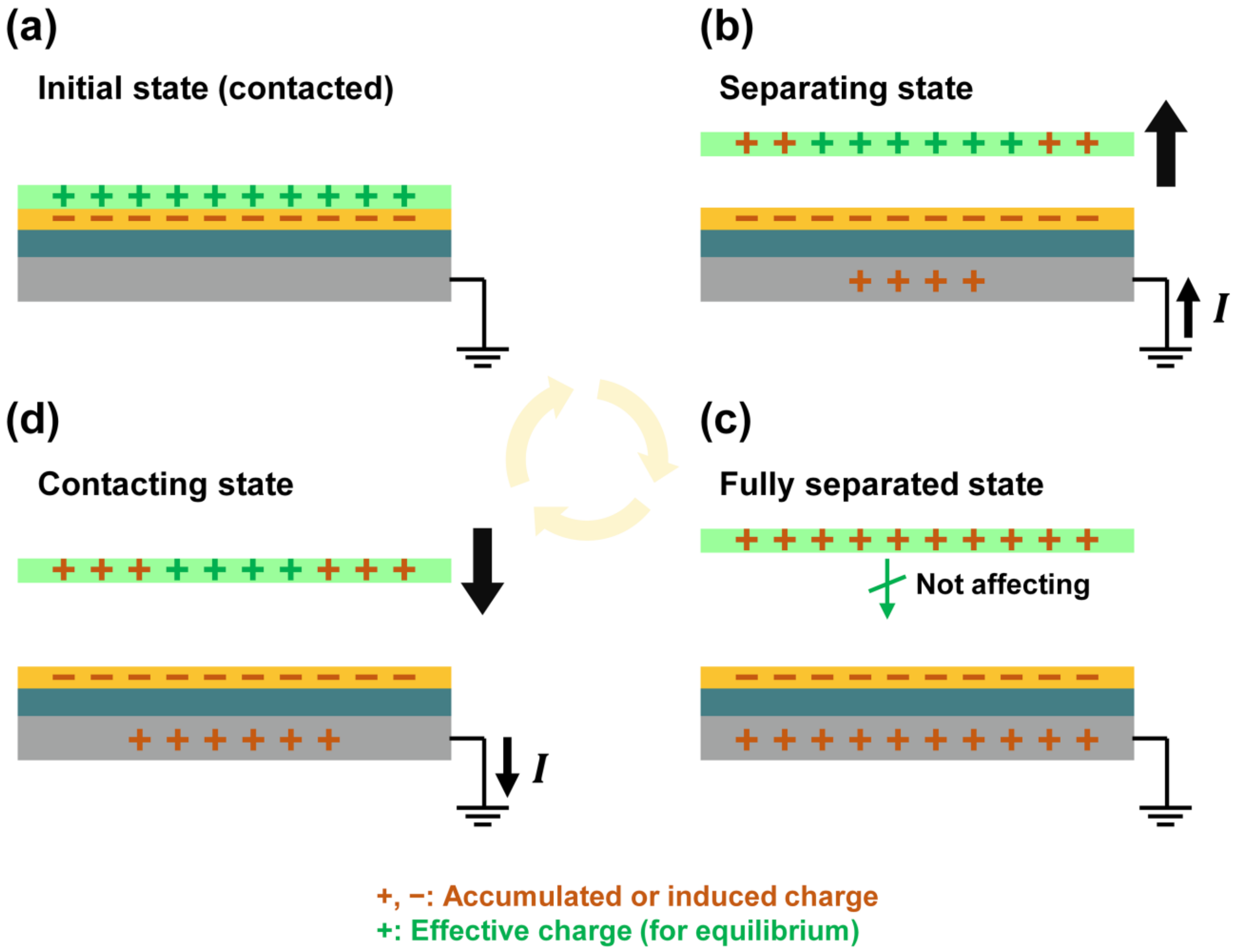

Figure S6. (a–d) Charge distribution profile in the TENG device while changing the contacting/separating states.

7. Fabrication process for $CaCO_3$-based TENG device

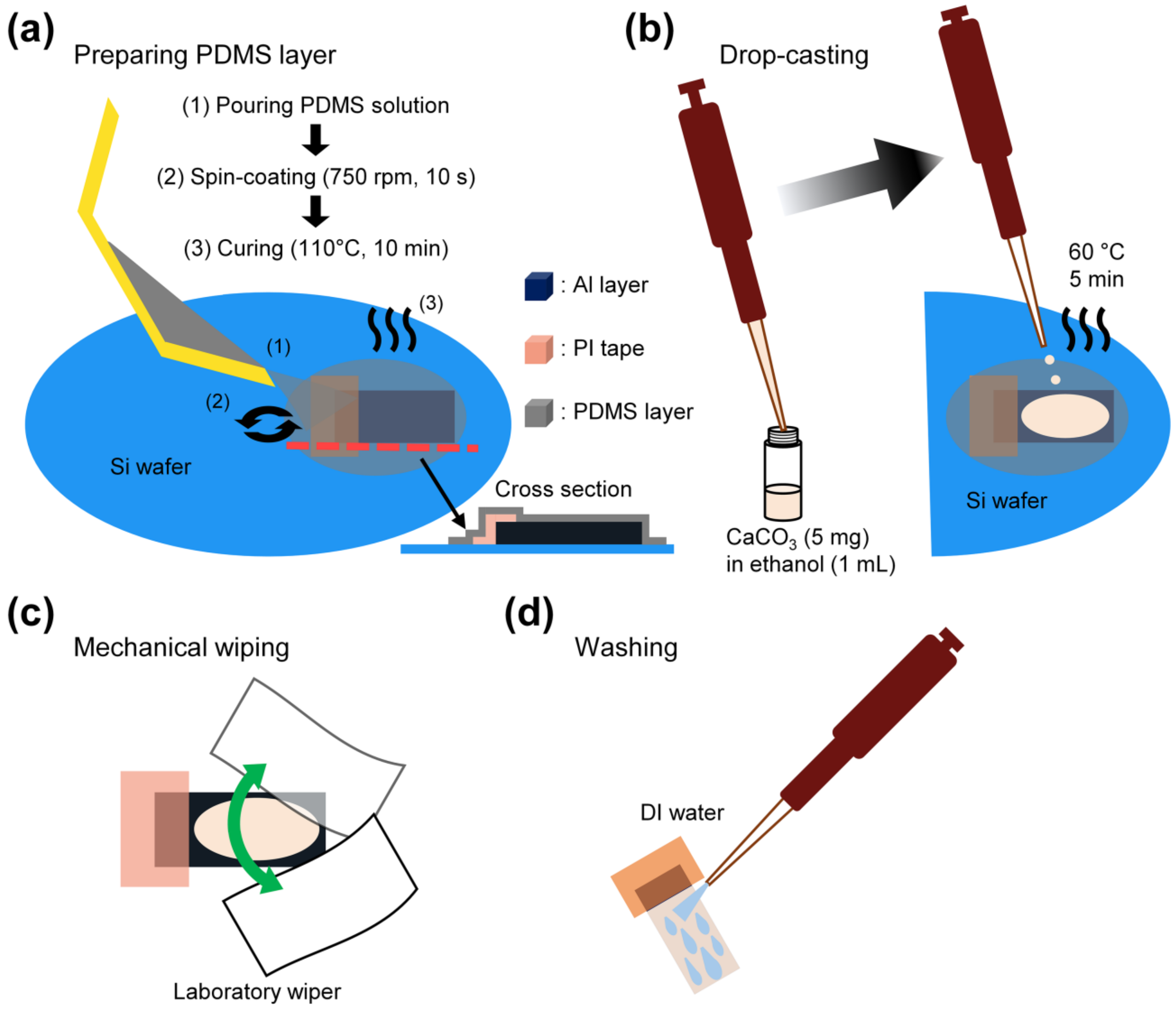

Figure S7. Fabrication process of the $CaCO_3$-based TENG. (a) Formation of polydimethylsiloxane (PDMS) layer. (b) Drop casting the optimized $CaCO_3$ dispersion on the PDMS layer. (c) Mechanical wiping process for uniformly distributing the $CaCO_3$ powder on the PDMS layer. (d) Washing process for removing weakly attached $CaCO_3$ particles.

8. Size distribution of the $CaCO_3$ particles with four different concentrations of dispersion

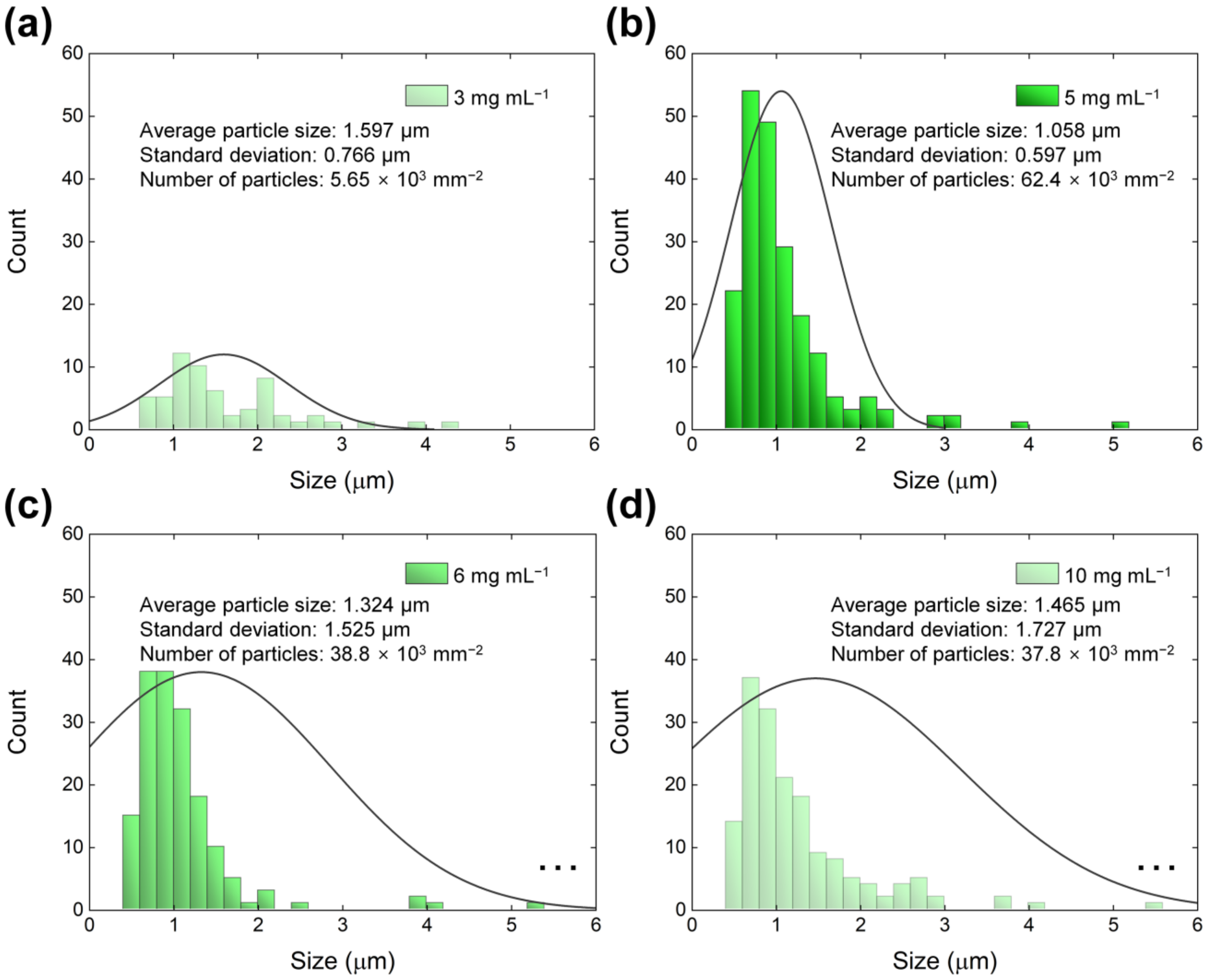

Figure S8. $CaCO_3$ particle size distribution with the dispersion-concentration values of (a) 3, (b) 5, (c) 6, and (d) 10 mg mL^{-1}.

9. Dispersion stability result of the $CaCO_3$ powder in ethanol

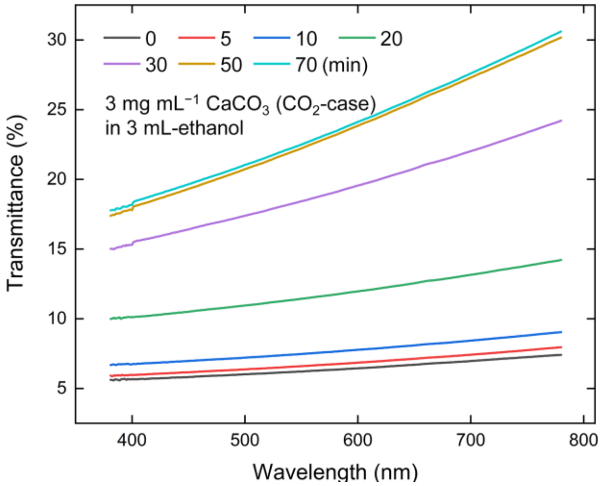

Figure S9. UV-vis spectra with the $CaCO_3$ dispersion of 3 mg mL^{-1} concentration with varying the stabilization time.

10. I_{SC} curves to check the triboelectric polarity of $CaCO_3$

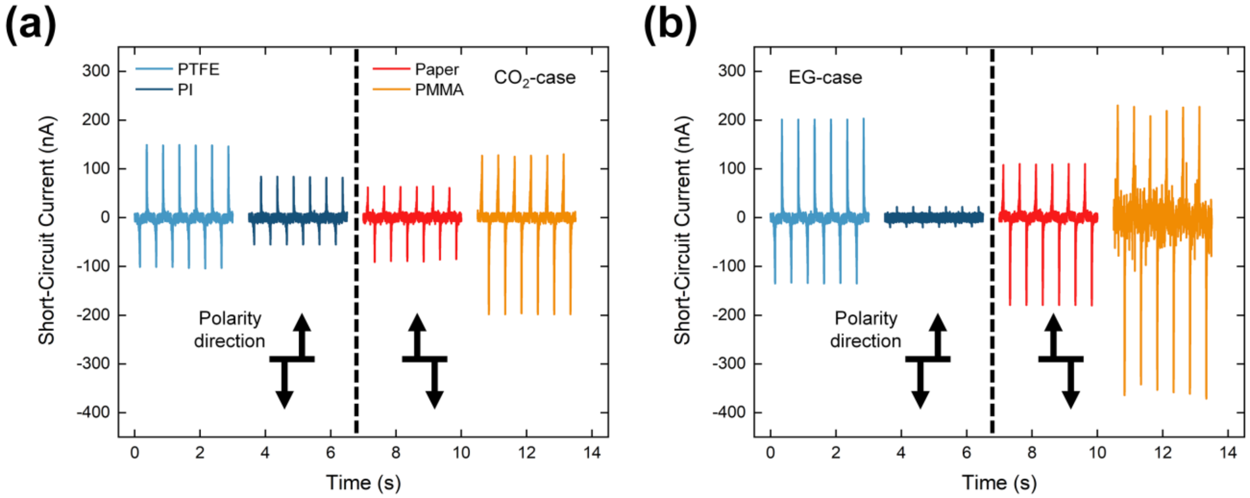

Figure S10. Measured I_{SC} results from the $CaCO_3$-based TENGs with the (a) CO_2-case and (b) EG-case while changing the counter triboelectric materials.

11. Fabrication process of eggshell-based $CaCO_3$ and electrical outputs using this powder

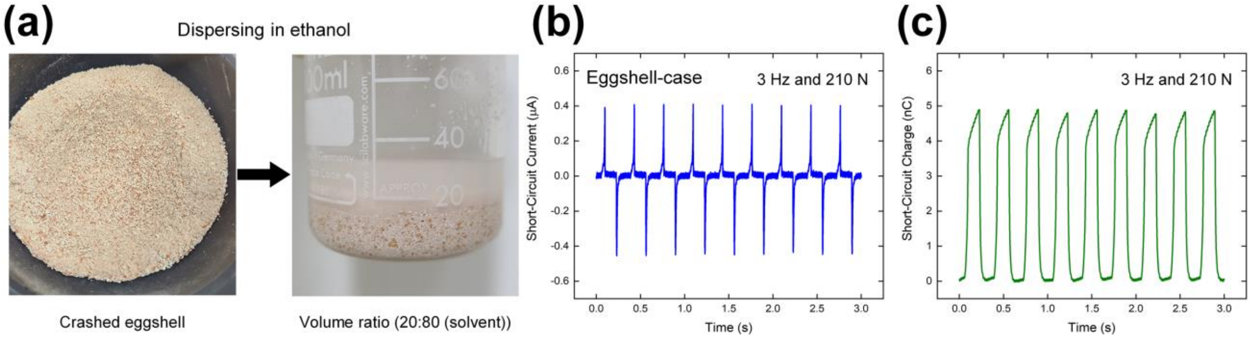

Figure S11. (a) Fabrication process of the eggshell-based $CaCO_3$ powder. (b) I_{SC} and (c) Q_{tr} of the TENG with the contact of the eggshell-based $CaCO_3$ layer and the paper layer.

12. Electrical output-response results from $CaCO_3$-based TENG with EG-case

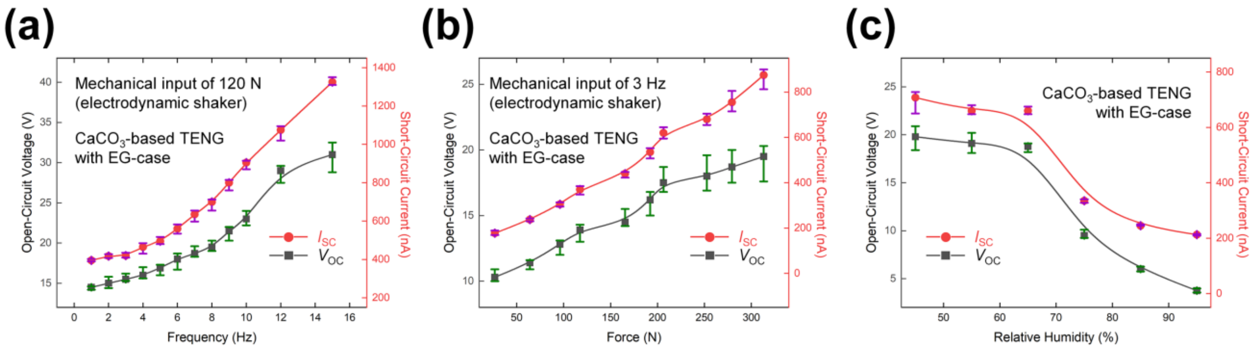

Figure S12. (a) Frequency response, (b) force response, and (c) humidity response results from the $CaCO_3$-based TENG with the EG-case.

13. Output current and power curves of two $CaCO_3$-based TENGs

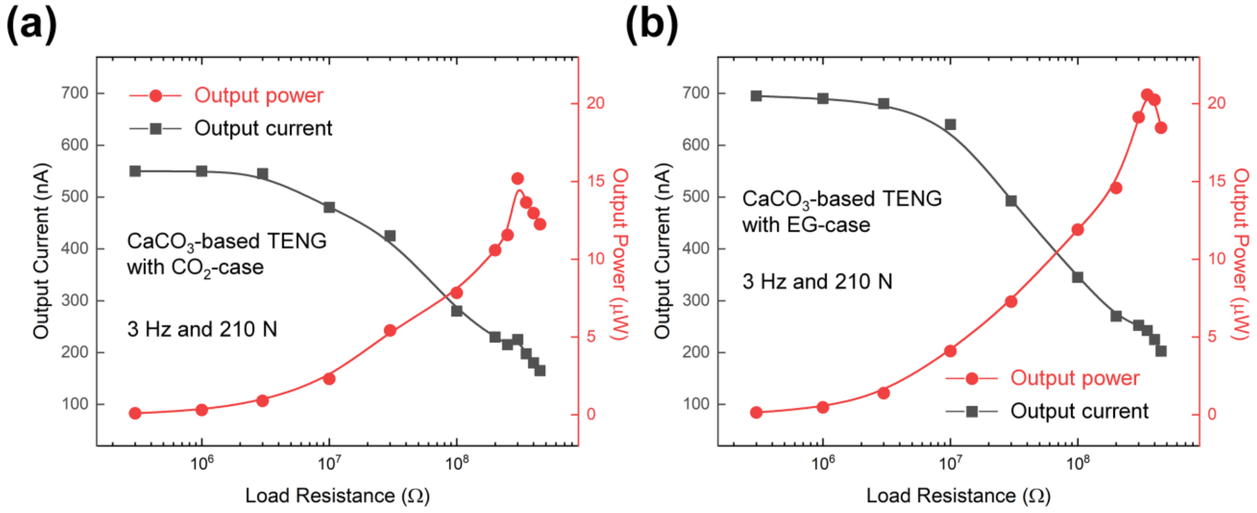

Figure S13. Electrical output current and power curves of the two $CaCO_3$-based TENGs with the (a) CO_2-case and (b) EG-case.

14. Durability result of TENG with a bare PDMS layer

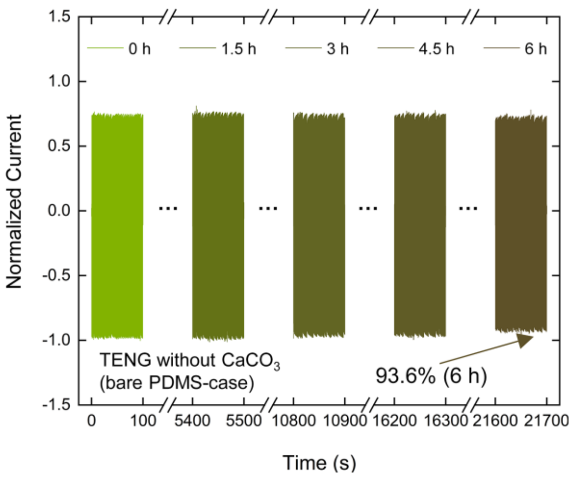

Figure S14. Normalized output current from the TENG without $CaCO_3$ layer for 21,700 s of operation.

15. Surface and size distribution of the $CaCO_3$ particles after measurement

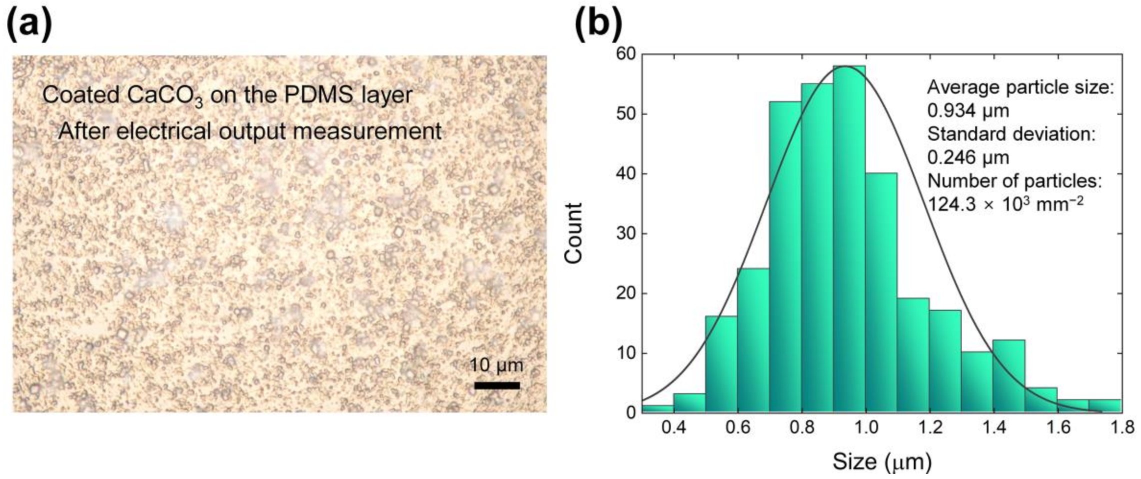

Figure S15. (a) OM image for the $CaCO_3$ particles and (b) size distribution profile of the $CaCO_3$ particles on the PDMS layer.

16. Fabrication process of Ddakji for slap match application

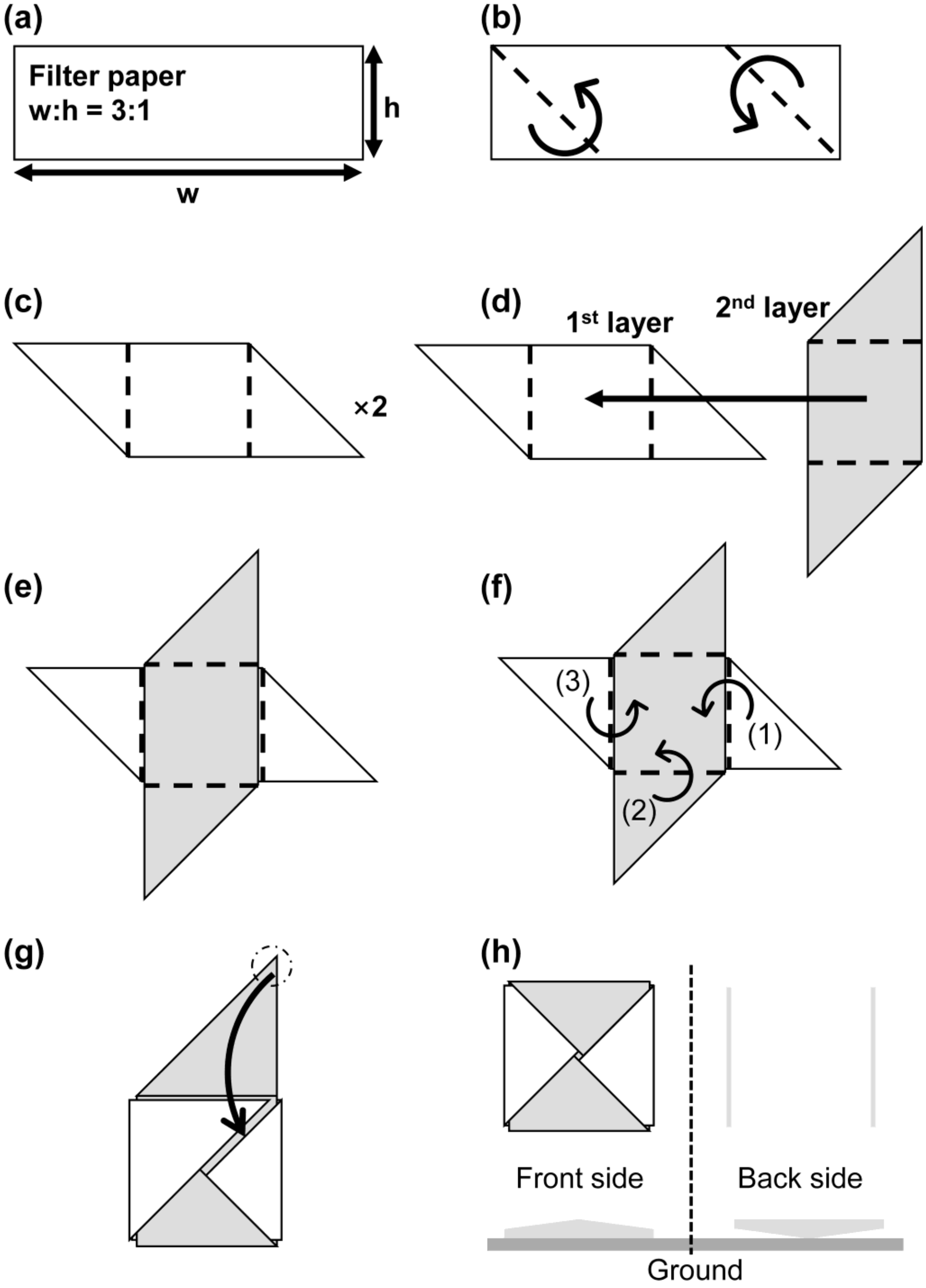

Figure S16. (a–h) Fabrication process of Ddakji based on paper work (origami).

17. Video of overturn case in slap match game

Video S1. Overturn case of the defender Ddakji by hitting the center part of the impact sensor array and corresponding peaks of the output voltage.

18. Video of unturn case in slap match game

Video S2. Unturn case of the defender Ddakji by hitting the side part of the impact sensor array and corresponding peaks of the output voltage.
